# Uptake and factors that influence the use of ‘sit less, move more’ occupational intervention strategies in Spanish office employees

**DOI:** 10.1186/s12966-014-0152-6

**Published:** 2014-12-10

**Authors:** Judit Bort-Roig, Montserrat Martin, Anna Puig-Ribera, Ángel Manuel González-Suárez, Iván Martínez-Lemos, Joan Carles Martori, Nicholas D Gilson

**Affiliations:** Grup de Recerca en Esport i Activitat Física, Universitat de Vic-Universitat Central de Catalunya (UVic-UCC), c/ Sagrada Família 7, 08500 Vic, Spain; Departamento de Educación Física y Deportiva, Universidad del País Vasco, Portal de Lasarte 71, 01007 Vitoria-Gasteiz, Spain; Facultad CC.EE. do Deporte, Universidad de Vigo, Campus A Xunqueira s/n, 36005 Pontevedra, Spain; Grup de Recerca DAM (Data Analysis and Modeling), Universitat de Vic-Universitat Central de Catalunya (UVic-UCC), c/Sagrada Família 7, 08500 Vic Barcelona, Spain; The University of Queensland, School of Human Movement Studies, St. Lucia Campus, 4072 Brisbane, Australia; Centre d’Estudis Sanitaris i Socials, Universitat de Vic-Universitat Central de Catalunya (UVic-UCC), c/ Miquel Martí i Pol, 1 08500 Vic, Spain

**Keywords:** Workplace, Occupational sitting, Sedentary behaviour, Walking, Multi-method study, Employee experiences

## Abstract

**Background:**

Little is known about the types of ‘sit less, move more’ strategies that appeal to office employees, or what factors influence their use. This study assessed the uptake of strategies in Spanish university office employees engaged in an intervention, and those factors that enabled or limited strategy uptake.

**Methods:**

The study used a mixed method design. Semi-structured interviews were conducted with academics and administrators (n = 12; 44 ± 12 mean SD age; 6 women) at three points across the five-month intervention, and data used to identify factors that influenced the uptake of strategies. Employees who finished the intervention then completed a survey rating (n = 88; 42 ± 8 mean SD age; 51 women) the extent to which strategies were used [never (1) to usually (4)]; additional survey items (generated from interviewee data) rated the impact of factors that enabled or limited strategy uptake [no influence (1) to very strong influence (4)]. Survey score distributions and averages were calculated and findings triangulated with interview data.

**Results:**

Relative to baseline, 67% of the sample increased step counts post intervention (n = 59); 60% decreased occupational sitting (n = 53). ‘Active work tasks’ and ‘increases in walking intensity’ were the strategies most frequently used by employees (89% and 94% sometimes or usually utilised these strategies); ‘walk-talk meetings’ and ‘lunchtime walking groups’ were the least used (80% and 96% hardly ever or never utilised these strategies). ‘Sitting time and step count logging’ was the most important enabler of behaviour change (mean survey score of 3.1 ± 0.8); interviewees highlighted the motivational value of being able to view logged data through visual graphics in a dedicated website, and gain feedback on progress against set goals. ‘Screen based work’ (mean survey score of 3.2 ± 0.8) was the most significant barrier limiting the uptake of strategies. Inherent time pressures and cultural norms that dictated sedentary work practices limited the adoption of ‘walk-talk meetings’ and ‘lunch time walking groups’.

**Conclusions:**

The findings provide practical insights into which strategies and influences practitioners need to target to maximise the impact of ‘sit less, move more’ occupational intervention strategies.

## Introduction

Prolonged periods of sitting have been linked to a range of chronic conditions such heart disease, type II diabetes and obesity [1-3]. A recent meta-analysis using data from over half a million adults indicated a 5% increased risk of all-cause mortality for each incremental hour of sitting, in those who sat for more than seven hours/day [4]. Office employees spend much of their work-related time sitting at a desk for up to six hours/day [5], and are therefore particular exposed to the health risks of prolonged sitting.

Height adjustable sit-stand desks have been proposed as one strategy to reduce and break occupational sitting [6-8]. Yet, the energy costs of sitting and standing are similar [9,10]. While valuable for metabolic health [11,12] such desks may therefore be less suitable for the prevention and management of weight related issues, because they do not raise energy expenditure across the working day.

Comprehensive intervention strategies that encourage moving as well as standing are therefore needed. Numerous studies have evidenced the effectiveness of workplace walking programs [13-15], but even though an inverse relationship has been suggested to exist between sitting time and step counts [16], few studies have purposely targeted reductions in occupational sitting and increases in workplace walking. Two such studies, of a ‘sit less, move more’ web-based program termed *Walk@Work* (W@W), which utilises incidental, and sustained walking strategies, reported average reductions in sitting time of up to 20 minutes/day and increases in walking in the most inactive of around 2,000 steps/day [14,17]. However, intra and inter individual variability in sitting and walking changes were evident across the program, suggesting that combination strategies were effective in some, but not all employees.

Furthermore, the emerging intervention evidence base concerning occupational sitting reduction strategies has tended to focus on outcome measures [14,15,18,19], rather than comprehensive process evaluation of intervention effectiveness. Grunseit et al. [20] and Chau et al. [21] have conducted interviews and focus groups respectively, among employees after using sit-stand desks, and authors concluded that sit stand desks had high usability and acceptability between users, identifying some barriers (e.g. issues with sit-stand workstation design) and facilitators (e.g. perceived health and work benefits). Nonetheless, very few studies have examined participants’ perceptions of ‘sit less, move more’ strategies [22,23].

Recognising these gaps in the extant literature, and using data from a subsequent program which implemented W@W in Spanish employees (W@WS; 2010–11), this study aimed to assess the uptake of strategies to reduce sitting time and increase walking at work. The study also explored factors that enabled or limited uptake of strategies to inform ongoing intervention efforts.

## Methods

### Study design

The study adopted a multi-strand parallel design, which combines qualitative and quantitative approaches at different study stages, [24] to better explore enablers and barriers that may have influenced the uptake of ‘sit less and move more’ strategies throughout the intervention process. Participants (n = 129) were self reported inactive (<3,000 MET · min · wk-1; IPAQ Short Form [25]) administrative and academic employees from four universities in the Spanish regions of Galicia, the Basque Country and Catalonia. The study was approved by the following ethics committee of each university: Ethics Committee of the Faculty in Psychology, Education and Sport Sciences (University Ramon Llull); Research Commission of University of Vic; Ethics Committee of Clinical Research in Conselleria de Sanidad (CEIC; Xunta de Galicia); Ethics Committee of Applied Research in Human Beings (CEISH/GIEB; University of the Basque Country).

### The W@WS program

W@WS is an automated web-based program which aims to encourage office employees to progressively ‘sit less and move more’ during workdays. The Spanish program is based on previous W@W initiatives, but implemented over a longer duration (19 weeks compared to 6 weeks), with an additional intervention stage designed to elicit increases in walking intensity. Table 1 describes the specific W@WS strategies used at different intervention stages. The first two weeks target breaking occupational sitting time through incidental movement during work tasks (Phase I). Subsequent weeks build on this ‘small changes’ approach by reducing overall sitting time through short walks (5–10 minutes), during morning/afternoon work breaks and/or commuting time (Phase II; weeks 3–4); and longer walks (10 minutes or more) at lunchtime (Phase III; weeks 5–6)*.* During weeks 7–8 (Phase IV), employees are presented with the aim of regularly achieving at least 10,000 daily steps, and also encouraged to increase walking intensity. An 11-week maintenance period then provides automated guidance with periodic emails encouraging workers to sustain changes in sitting and walking, achieved in previous phases.

**Table 1:** **W@WS ‘sit less and move more’ intervention strategies relative to program phases**

**Stages**	**Aim**	**Strategies**
Incidental movement	Integrate incidental movement into work tasks.	Take advantage of centralized office equipment (e.g. photocopier or printer) and spread these work tasks out through the day.
Phase I (weeks 1–2)		
		When agreed and appropriate with colleagues, deliver some messages in person, rather than always sending emails.
		Stand up and/or move around the office while talking on the phone or reading documents.
		When appropriate, organise walk-talk, rather sit-talk meetings.
Short walks	Implement short, regular walks of 5–10 minutes at opportune times across the work day.	Active morning and afternoon work breaks.
Phase II (weeks 3–4)		Active travel during commuting (e.g. park the car further and walk, or walk and take public transport).
Longer walks	Undertake a longer, daily walk of 10 minutes or more during the working week.	Organise walks with colleagues, or plan to walk alone, at lunch time or before/after work.
Phase III (weeks 5–6)		
Higher step count frequency and intensity	Regularly achieve 10,000 daily steps and raise the intensity of some short and longer walks.	Identify opportunities to increase the frequency of incidental movement and short/longer walks.
Phase IV (weeks 7–8)		When moving around the workplace, use the stairs instead of lifts or escalators.
		Use the natural environment and plan longer walking routes that include inclines or steps.
		Increase step cadence, or the number of steps taken each minute on short and longer walks.

Program participants use a pedometer (Yamax SW-200) and diary to self-report daily step counts and sitting time (hours and minutes of daily sitting) in conjunction with the W@WS website, which provides a range of ecological support strategies to facilitate sitting time reductions and step count increases at work. This includes logging daily step counts into a personal account and receiving feedback on the achievement of goals through visual graphics and prompts. Furthermore, the website provides tips for achieving the targets in each phase, social networking for sharing experiences, and educational materials on the health benefits of ‘sitting less and moving more’.

### Interviews

The qualitative strand of the study occurred through semi-structured interviews, carried out at the end of the phase II, phase IV, and after the maintenance phase (with four interviews in each phase to achieve data saturation). We choose interviews over focus groups, to provide detailed and in depth insights into individual employee experiences at multiple points across the program. We aimed to recruit 12 employees for interviews from the W@WS sample, with four employees from each region (Galicia, Basque Country and Catalonia). To capture different viewpoints, heterogenic selection criteria were used, relative to baseline inactivity level, gender and job role. Beginning from the least active at baseline, male and female academics and administrators were approached by email individually until these categories were represented within each region.

Semi-structured interviews were undertaken with the purpose of exploring factors that were perceived to enable or limit the implementation of strategies to reduce sitting and increase walking. Interviews used open ended questions which captured, at each relevant period of the intervention, a) employee motivations and personal expectations for program involvement; b) the types of strategies adopted or discarded and; c) the reasons why this was the case (i.e. factors that enabled or limited strategy uptake).

Interviews lasted for around 40–60 minutes and were conducted in Catalan or Spanish. Employee responses were audio recorded, then fully transcribed and subjected to inductive open coding to identify emerging categories. Two researchers performed analyses independently, and then met to discuss and agree key themes. Employee quotes to support themes were identified and then back translated from Catalan/Spanish to English.

### Post intervention surveys

The quantitative strand of the study involved administering a survey to all employees who completed the W@WS program, two months following intervention (n = 88). The survey contained seven items assessing the uptake of each strategy described, with response options ranging from 1 (never) to 4 (usually). An additional 17 items assessed factors that enabled (9 items) and limited (8 items) the uptake of strategies; response options for these items ranged from 1 (no influence) to 4 (highly influential). Survey items assessing enabling and limiting factors were developed using the thematic outcomes from the qualitative study strand, and reviewed independently by three researchers to establish face validity.

Demographic (age, gender, BMI and job role), and behaviour data (self-reported sitting by diary logging and pedometer derived step counts), collected as part of the main W@WS program, were used to describe the characteristics of interviewees and those employees who completed the survey. Mean and proportion item scores for the surveys were analysed to report factor distributions. Quantitative findings were triangulated with interview data to provide comprehensive, mixed method insights into participant experience.

## Results

Table 2 describes the demographic and behavioural characteristics of interviewees and survey participants. Those in both samples tended to be middle aged employees (42–44 years), approaching or just over the overweight BMI threshold (24.9-25.4 kg/m^2^). Survey participants were relatively evenly split between academics and administrators, although two thirds were women. At baseline, employees averaged between 6,000 – 8,000 daily steps and 7–9 hours/day sitting; 60% and 67% of employees who completed W@WS decreased sitting time and increased step counts respectively.

**Table 2:** **Interviewee and survey participant demographics at baseline, and sitting time and step count changes post intervention**

	**Interviewees (n = 12)**	**Survey (n = 88)**
Age	44 ± 12 years	42 ± 8 years
Gender		
Men	n = 6	n = 35 (39%)
Women	n = 6	n = 53 (61%)
Job role		
Academic	n = 6	n = 52 (59%)
Administrative	n = 6	n = 37 (41%)
Body Mass Index (BMI)	24.9 ± 2.8 kg/m^2^	25.4 ± 4.0 kg/m^2^
Walking		
Baseline	6,800 ± 1,844 steps/day	8,788 ± 2,691 steps/day
Number who increased	n = 10 (87%)	n = 59 (67%)
Sitting		
Baseline	8.8 ± 1.8 hours/day	7.4 ± 2.2 hours/day
Number who decreased	n = 4 (37%)	n = 53 (60%)

Data presented as mean ± SD for continuous variables and n (%) for categorical variables.

### Uptake of strategies

Post-intervention survey data at two months follow up (Table 3) examined the uptake of ‘sit less, move more’ strategies. ‘Active work tasks’ (incidental movement) and ‘stairs, natural inclines and step count cadence’ (higher intensity walking) were the most popular in terms of uptake, with a high percentage of employees reporting that they usually or sometimes used these strategies during the program (89-94% respectively).

**Table 3:** **Number (%) of employees using W@WS strategies and survey score averages at two months follow up**

**Strategies**	**Never (1)**	**Hardly ever (2)**	**Sometimes (3)**	**Usually (4)**	**Survey score (Mean ± SD)**
Incidental movement (phase I: weeks 1–2)					
Active work tasks (e.g. using a centralized Printer or active emails)	2 (3)	7 (8)	47 (56)	28 (33)	3.1 (0.7)
Walk-talk meetings	48 (59)	17 (21)	9 (11)	7 (9)	1.7 (1.0)
Short walks (phase II: weeks 3–4)					
Active work breaks	15 (18)	20 (24)	36 (44)	11 (14)	2.5 (0.9)
Active travel during commuting	23 (29)	9 (12)	15 (19)	31 (40)	2.7 (1.3)
Longer walks (phase III: weeks 5–6)					
Groups	70 (83)	11 (13)	2 (3)	1 (1)	1.2 (0.5)
Alone	26 (30)	24 (30)	22 (27)	11 (13)	2.2 (1.0)
Higher intensity walking (phase IV: weeks 7–8)					
Stairs, natural inclines and step cadence	2 (2)	3 (4)	22 (27)	55 (67)	3.6 (0.7)

Moderately used strategies were ‘active work breaks’ and ‘active travel during commuting’. Around 60% of employees reported sometimes or usually using these short walk approaches.

‘Walking alone’ (longer walks) and ‘walk-talk meetings’ (incidental movement) were reported to be hardly ever or never used by the majority of employees (60-80% respectively). ‘Walking in groups’ (longer walks), was the least popular strategy in terms of uptake, with 96% of employees reporting that they hardly ever or never used this type of approach.

### Factors that enabled uptake

Qualitative analyses of interviewee data identified two sets of enablers (Table 4) facilitating uptake across strategies. Program supports provided through the W@WS website were a strong theme highlighted by all employees. Supports included web-based automated features that provided access to educational materials and visual representation of progress through graphics. For example two interviewees reported that:

**Table 4:** **Factors that enabled strategy uptake: survey score distributions (number of employees and [%]) and averages**

**Facilitators**	**No influence (1)**	**Some influence (2)**	**Strong influence (3)**	**Very strong influence (4)**	**Survey score (Mean ± SD)**
Program supports					
Pedometer and diary logging	2 (3)	16 (19)	38 (45)	28 (33)	3.1 (0.8)
Educational materials	8 (10)	18 (21)	43 (51)	15 (18)	2.8 (0.9)
Following an aim for each phase	9 (11)	18 (21)	42 (50)	15 (18)	2.8 (0.9)
Following progression by visual graphics	8 (10)	39 (46)	26 (31)	11 (13)	2.5 (0.8)
Receiving fortnightly emails	16 (19)	47 (56)	21 (25)	-	2.1 (0.7)
Health-related work context					
Being aware of too much occupational sitting	2 (2)	27 (32)	36 (43)	19 (23)	2.9 (0.8)
Being aware of opportunities to ‘sit less and move more’ at work	4 (5)	22 (26)	44 (52)	14 (17)	2.8 (0.9)
Percieved improvements in health	17 (21)	40 (49)	20 (24)	5 (6)	2.2 (0.8)
Previous or current health conditions	55 (65)	17 (21)	11 (13)	1 (1)	1.5 (0.8)

*“An important factor has been the information the program has provided for me. This has helped me to be aware of the benefits of being less sedentary and more active”.* (Interviewee 7; Female Academic)

*“Checking my global progress visually* [using the individualised graphs provided by the W@WS website] *has helped me to be more motivated”.* [to sit less and move more (Interviewee 8; Female Administrator)

Interviewees also highlighted the value of receiving regular fortnightly emails, logging steps and sitting into a personal diary, and following a goal for each phase. These factors were evidenced by three employees who indicated that:

*“The messages [the fortnightly emails] I receive regularly are very valuable. They encourage me to persevere. The overall message is clear: Keep going!”* (Interviewee 1. Male Academic)

*“At the end of the day when you are recording the hours you’ve been seated during work time and the number of steps, you realise how sedentary you’ve been. This is an extra motivation for the next day to try to move more”.* (Interviewee 5. Male Academic)

*“Keeping* [the program] *goals in mind helps you … you try to achieve and surpass these goals*.” (Interviewee 12. Female Administrator)

A second set of enablers were themed under the area of work context and health. The majority of interviewees (11 employees), specifically mentioned being aware of spending too many hours sitting at work. This awareness encouraged them to take part in the W@WS program and follow the strategies to change this behaviour. Linked to this, nine employees mentioned that program strategies raised awareness of how to implement ‘sit less and move more’ approaches into the working day. As the following quotes illustrate:

*“Because of the nature of the job, I’m sitting all day.... When I saw the program I thought it would be an opportunity for me* [to sit less at work]*”.* (Interviewee 1; Male Academic)

*“Sometimes I realise that what I am doing* [at work] *I could do walking up and down, for instance when I have to read a document…”* (Interviewee 10; Male Administrator)

*“If I have to go from A to B, I do a little detour and I pass though C, instead of it being 5 minutes it takes me 10 minutes… I take advantage of my time”.* (Interviewee 11; Female Academic)

Four interviewees directly attributed their health issues (e.g. back pain or hypertension) with their sedentary work context. Therefore, having a health condition that could benefit from intervention was perceived as enabling (as opposed to limiting) the uptake of strategies. As one of these interviewees contested:

*“I have high cholesterol and the doctor told me I had to do something, then I thought that this program could be a very good way for me to fix it”.* (Interviewee 6; Male Administrator)

Concurrent with this idea, ten interviewees highlighted that they experienced improvements in physical and mental health through the W@WS program. For example:

*“Now after lunch, instead of going to have a coffee next to my office I go to the main square in town… then I get out of the Uni,… it’s not only about doing more steps, it’s about mental relaxation as well”.* (Interviewee 3; Female Academic)

*“Before when I climbed up the stairs I used to get out of breath. Now when I take the steps to the 3*^*rd*^*floor I’m not out of breath; I could even go up one more floor! This increases my self esteem and encourages me to keep going with the program”.* (Interviewee 1; Male Academic)

Survey data which built on qualitative themes and captured level of influence across the W@WS sample, indicated that ‘pedometer and diary logging’ was perceived to be the most important enabler (mean survey score of 3.1 ± 0.8); 78% of employees rated this factor as a strong or very strong influence. Conversely, ‘previous or current health conditions’ was considered to be the least important enabler (mean survey score of 1.5 ± 0.8); 65% of employees reported this factor to be uninfluential. Remaining enablers were influential to some degree along this continuum, with mean survey scores for factors ranging from 2.1-2.9, and composite influence percentages (categories 2–4) ranging from 81-98%.

### Factors that limited uptake

Thematic analyses of interviewee data identified eight factors that limited uptake of W@WS strategies (Table 5). Two of these factors, identified by six interviewees, described generalised barriers such as poor goal setting, and not being able to accurately estimate the amount of time sitting at work. As three of these employees stated:

**Table 5:** **Factors that limited strategy uptake: survey score distributions (number of employees and [%]) and averages**

**Barriers**	**No influence (1)**	**Some influence (2)**	**Strong influence (3)**	**Vey strong influence (4)**	**Survey score (Mean ± SD)**
Screen based work	3 (4)	13 (15)	32 (38)	36 (43)	3.2 (0.8)
Lack of time, time pressure, and excessive workload	12 (14)	16 (19)	27 (32)	29 (35)	2.9 (1.1)
Not being fully aware of the amount of time spent sitting at work	19 (23)	31 (37)	23 (28)	10 (12)	2.3 (1.0)
Bad weather	26 (31)	34 (41)	17 (20)	7 (8)	2.1 (0.9)
Lack of support from colleagues	45 (56)	15 (19)	12 (15)	8 (10)	1.8 (1.0)
Poor goal setting	40 (48)	31 (37)	11 (13)	2 (2)	1.7 (0.8)
Lack of support from management team	57 (69)	11 (13)	9 (11)	6 (7)	1.6 (1.0)
Belief that physical activity outside work offsets long periods of sitting at work	50 (60)	22 (27)	11 (13)	-	1.5 (0.7)

*“I am unable to know how long I sit every day. I get up from the chair quite frequently, so it’s impossible”.* (Interviewee 12; Female Administrator)

*"I think it's more difficult to reduce sitting time than to increase walking… it’s difficult because we can’t count* [minutes spent sitting] *with the same accuracy as the number of steps".* (Interviewee 7; Female Academic)

*“I got frustrated with not getting to my target. I thought ‘fail, fail’ and ended up not achieving …”* (Interviewee 2; Male Administrator)

Other limiting factors were specifically linked to the strategies listed in Table 3. For example, for Phase I strategies, seven interviewees encountered difficulties in carrying out ‘active work tasks’ and ‘walk-talk meetings’, because colleagues did not perceive these strategies to be suitable for the workplace, or the nature of work involved (which mainly concerned sitting in front of the computer) limited the opportunity to move. As two academics commented:

*“… I sometimes send stuff to the printer and I walk a bit more around the office, but I don’t do it much because I think my colleagues would think I’m crazy”.* (Interviewee 7; Female Academic)

*“When I have a meeting with a student, I try to walk up and down, but it’s difficult because we need to write things down or work with the computer”.* (Interviewee 9; Male Academic)

Relative to Phase II strategies (short walks), interviewees reported time pressures and an excessive workload as factors that limited the uptake of active breaks. However, perceptions of impact for this factor tended to differ between occupational roles. This was attributed to the inherent flexibility in job tasks, where administrative employees reported to have a more structured (and supervised) schedule than academics, who within limits, could choose how and when to work. A recurring theme amongst interviewees was the perception that managers viewed administrator absenteeism from desks for walking unfavourably, whereas academics did not feel obliged to justify their absence for a walking break. As the following quote illustrates:

*“… I have my boss round the corner… I don’t have much excuse to move around”.* (Interviewee 12; Female Administrative)

Ten interviewees cited time pressures inside work as a key barrier limiting longer walks during Phase III. Having an active leisure time after work was viewed as a feasible alternative. For example:

*“I prefer to get the best out of my time in the office*, *and then to be able to leave a bit earlier and go to the gym….”* (Interviewee 5; Male Academic)

*“Because I sit for so long during my work hours, I compensate by walking after work”.* (Interviewee 8; Female Administrator)

However, four employees tried to find time during lunchtime for movement and highlighted this period of the day as being flexible enough to allow longer walks to occur regularly across the working week. Two employees disagreed with this viewpoint and did not find longer lunchtime walks appealing as it meant missing important social interactions with other workers. As one interviewee commented:

*“Walking at lunch time means… less social time with colleagues…”* (Interviewee 11; Female Academic)

Seven interviewees also highlighted inclement weather during wintertime as a factor limiting the uptake of longer walks. As one employee from the North of Spain highlighted:

*“Here we have very severe winters and do not want to move much. You sometimes have to go to another building and you get wet, with or without umbrella.”* (Interviewee 6; Male Administrator)

Post interview survey data identified ‘screen based work’ (i.e. sitting at a desk working on a computer) as the most influential barrier (mean survey score of 3.2 ± 0.8); 81% of employees reported this factor to be highly influential in limiting W@WS strategy uptake. ‘Lack of time, time pressure, and excessive workload’ , ‘not being fully aware of the amount of time spent sitting at work’ and ‘bad weather’ were rated as second tier influences; mean survey scores for these barriers ranged from 2.1-2.9, and 64-69% of employees suggested that these factors limited strategy uptake to some degree. The remaining four factors were lower level influences, with mean survey scores ranging from 1.5-1.8; ‘belief that physical activity outside work offsets long periods of sitting at work’ and ‘lack of support by management team’ were the lowest ranking factors in this group; 60-69% of employees classified these limiting factors identified by interviewees as uninfluential.

## Discussion

The aims of this study were twofold. Using a multi-method approach, we assessed the uptake of ‘sit less, move more’ strategies, and also explored factors that enabled and limited sitting time reductions and walking increases in Spanish university office employees from the W@WS project. Exploring the specific types of strategies that facilitated these changes was a novel and valuable aspect of the present study, particularly for health practitioners interested in gaining practical advice on how to maximize intervention efficacy.

Accordingly, the findings suggest that promoting workplace strategies that target ‘active work tasks, active work breaks, active travel, and higher step count frequency and intensity’ may appeal to office employees. Conversely, strategies that require employees to engage in walk-talk meetings or longer individual or group based lunchtime walking sessions may be less popular and therefore less effective at encouraging employees to reduce sitting time and increase step counts.

Investigating factors that enabled the uptake of intervention strategies was also a unique aspect of the present study. Using a pedometer and diary to report sitting time and step counts throughout the program was reported to be the most important factor enabling sitting and walking changes in the survey sample. Several studies highlight pedometer-based interventions and step count logging in particular as an effective means of motivating people to be more active [26-28]; our findings suggest that this may also be an effective approach for reducing occupational sitting. However our interview participants also raised concerns about the practicality and accuracy of logging sitting times, highlighting the role new technologies, such as smartphones, may play in this regard [29].

Similar to previous research [27,30], provision of educational materials and goal setting were also considered useful enablers of strategy uptake. However, in contrast to other research [28], email reminders did not facilitate strategy use.

Mixed method data identifying key influences that limited the use of specific strategies are particularly valuable for practitioners and employers interested in overcoming barriers that discourage active workplaces. For example, while the concept of integrating ‘active work tasks’ into the working day was highly used, ‘active work breaks and longer lunchtime walks’ were negatively influenced to some degree by lack of time, socio-cultural expectations and excessive workloads. Although the belief that physical activity outside of work offsets long periods of sitting at work was identified as possible barrier, this did not influence strategy uptake.

Based on our qualitative and quantitative data sedentary work tasks were highlighted as the principal barrier discouraging meaningful engagement with strategies. From one perspective this supports the importance of targeting workstation based strategies (such as sit-stand, or treadmill desks [31]). From another perspective, our findings highlight the value of employee reflexivity and the provision of different types of strategies that target a range of occupational sitting and moving contexts. A key recommendation emerging from our study therefore concerns the value of providing a ‘menu’ of ‘sit less, move more’ strategies that employees can choose from and fit within and around the day-to-day demands of the office work environment.

It is important to set study recommendations against the context of our sample, which was relatively small and consisted of academic and administrative employees from Spanish universities. Given that occupational sitting and walking data were similar to those observed in office workers from other countries who have used the W@W program, patterns of strategy use and enabling/limiting factors may also be similar. However, to best inform translational efforts, on going research should aim to investigate these issues in other occupational groups from different cultures.

Other factors that may have influenced study outcomes include timing of the administration of the survey; which strategies were used by employees prior to and during early intervention; and the fact that most of our interviewed employees (from 60% to 67%) reduced sitting and increased walking. This latter point highlights the value of accessing employees who were less successful at implementing behaviour change.

The present study also had a range of strengths which future work in the area should build upon, such as the use of a mixed method approach, which combined quantitative data, with rich and meaningful qualitative experiences in a real world office setting. Other studies that have targeted occupational sitting or walking have assessed employee experiences post program, relative to single strategy approaches such as sit-stand desks [20,21] or pedometer based, walking interventions [22,27]. Our study comprehensively explored uptake and influences in a number of ‘sit less’ and ‘move more’ approaches, throughout the intervention process. Consequently, the findings provide valuable employee insights across a broad range of strategies, as and when experiences were taking place.

## Conclusions

This mixed method study found that ‘higher intensity walking and active work tasks’ were the most frequently used intervention strategies to decrease occupational sitting and increase workplace walking in Spanish university office employees. ‘Walk-talk meetings and lunchtime walking groups’ were used the least. Key facilitators and barriers to strategy uptake included ‘sitting time and step count logging’ and ‘screen based work’ respectively, with these data providing insights into which influences practitioners need to target to encourage employees to ‘sit less and move more’ at work.

## References

[1] Thorp A, Owen N, Neuhaus M, Dunstan DW (2011). Sedentary behaviors and subsequent health outcomes in adults a systematic review of longitudinal studies, 1996–2011. Am J Prev Med.

[2] Yates T, Khunti K, Wilmot EG, Brady E, Webb D, Srinivasan B, Henson J, Talbot D, Davies MJ (2012). Self-reported sitting time and markers of inflammation, insulin resistance, and adiposity. Am J Prev Med.

[3] Ford ES, Caspersen CJ (2012). Sedentary behaviour and cardiovascular disease: a review of prospective studies. Int J Epidemiol.

[4] Chau JY, Grunseit AC, Stamatakis E, Brown WJ, Matthews CE, Bauman AE, van der Ploeg HP (2013). Daily sitting time and all-causes mortality: A meta-analysis. PLoS One.

[5] Ryde GC, Helen EB, Peeters GME, Gilson ND, Brown WJ (2013). Desk-Based Occupational Sitting Patterns. Am J Prev Med.

[6] Gilson ND, Suppini A, Ryde GC, Brown HE, Brown WJ (2012). Does the use of standing “hot” desks change sedentary work time in an open plan office?. Prev Med.

[7] Straker L, Abbott RA, Heiden M, Mathiassen SE, Toomingas A (2013). Sit-stand desks in call centres: associations of use and ergonomics awareness with sedentary behavior. Appl Ergon.

[8] Neuhaus M, Healy GN, Fjeldsoe BS, Lawler S, Owen N, Dunstan DW, LaMontagne AD, Eakin EG (2014). Iterative development of Stand Up Australia: a multi-component intervention to reduce workplace sitting. Int J Behav Nutr Phys Act.

[9] Speck RM, Schmitz KH (2011). Energy expenditure comparison: a pilot study of standing instead of sitting at work for obesity prevention. Prev Med.

[10] Tudor-Locke C, Schuna JJ, Frensham L, Proenca M (2014). Changing the way we work: elevating energy expenditure with workstation alternatives. Int J obesity.

[11] Hamilton MT, Healy GN, Dunstan DW, Zderic T, Owen N (2008). Too little exercise and too mjuch sitting: Inactivity physicology and the need for new recommendations on sedentary behaviour. Curr Cardiovasc Risk Rep.

[12] Dunstan DW, Kingwell BA, Larsen R, Healy GN, Cerin E, Hamilton MT, Shaw JE, Bertovic DA, Zimmet PZ, Salmon J, Owen N (2012). Breaking Up Prolonged Sitting Reduces Postprandial Glucose and Insulin Responses. Diabetes Care.

[13] Chan CB, Ryan DAJ, Tudor-Locke C (2004). Health benefits of a pedometer-based physical activity intervention in sedentary workers. Prev Med.

[14] Gilson ND, Puig-ribera A, Mckenna J, Brown WJ, Burton NW, Cooke CB (2009). Do walking strategies to increase physical activity reduce reported sitting in workplaces: a randomized control trial. Int J Behav Nutr Phys Act.

[15] Freak-poli R, Wolfe R, Backholer K, De Courten M, Peeters A (2011). Impact of a pedometer-based workplace health program on cardiovascular and diabetes risk profile. Prev Med.

[16] Tudor-locke C, Craig CL, Thyfault JP, Spence JC (2013). A step-defined sedentary lifestyle index: < 5000 steps/day. Appl Physiol Nutr Metab.

[17] Gilson ND, Faulkner G, Murphy MH, Umstattd Meyer MR, Washington T, Ryde GC, Arbour-Nicitopoulos KP, Dillon KA (2013). Walk @ Work: An automated intervention to increase walking in university employees not achieving 10, 000 daily steps. Prev Med.

[18] Healy GN, Eakin EG, Lamontagne AD, Owen N, Winkler EAH, Wiesner G, Gunning L, Neuhaus M, Lawler S, Fjeldsoe BS, Dunstan DW (2013). Reducing sitting time in office workers: short-term efficacy of a multicomponent intervention. Prev Med.

[19] Chau JY, Daley M, Dunn S, Srinivasan A, Do A, Bauman AE (2014). The effectiveness of sit-stand workstations for changing office workers ’sitting time: results from the Stand @ Work randomized controlled trial pilot. Int J Behav Nutr Phys Act.

[20] Grunseit AC, Chau JY-Y, van der Ploeg HP, Bauman A (2013). “Thinking on your feet”: A qualitative evaluation of sit-stand desks in an Australian workplace. BMC Public Health.

[21] Chau JY, Daley M, Srinivasan A, Dunn S, Bauman AE, van der Ploeg HP (2014). Desk-based workers ’ perspectives on using sit-stand workstations: a qualitative analysis of the Stand @ Work study. BMC Public Health.

[22] Gilson N, Mckenna J, Cooke C (2008). Experiences of route and task-based walking in a university community: qualitative perspectives in a randomized control trial. J Phys Act Health.

[23] Cooley D, Pedersen S, Mainsbridge C (2014). Assessment of the impact of a workplace intervention to reduce prolonged occupational sitting time. Qual Health Res.

[24] Greene J, Carcelli V, Tashakkori A, Teddlie C (2003). Making paradigmatic sense of Mixed Methods practice. Hanbook Mixed Methods in social and behavioral research.

[25] Craig CL, Marshall AL, Sjo M, Bauman A, Booth ML, Ainsworth BE, Pratt M, Ekelund U, Yngve A, Sallis JF, Oja P (2003). International physical activity questionnaire : 12-country reliability and validity. Med Sci Sports Exerc.

[26] Gardner P, Campagna P (2011). Pedometers as measurement tools and motivational devices: new insights for researchers and practitioners. Heal Promot Pr.

[27] Lauzon N, Chan CB, Myers AM (2008). Participant experiences in a workplace pedometer-based physical activity program. J Phys Act Health.

[28] Heesch K, Dinger M, McClary K, Rice K (2005). Experiences of women in a mini- mal contact pedometer-based intervention: a qualitative study. Women Health.

[29] Bort-Roig J, Gilson ND, Puig-Ribera A, Contreras RS, Trost SG (2014). Measuring and influencing physical activity with Smartphone technology: a systematic review. Sport Med.

[30] Fukuoka Y, Lindgren T, Jong SS (2012). Qualitative exploration of the acceptability of a mobile phone and pedometer-based physical activity program in a diverse sample of sedentary women. Public Health Nurs.

[31] Neuhaus M, Eakin EG, Straker L, Owen N, Dunstan DW, Reid N, Healy GN (2014). Reducing occupational sedentary time: a systematic review and meta-analysis of evidence on activity-permissive workstations. Obes Rev.

